# Low Cognitive Performance Increases The Risk Of Hospital-Associated Complications In Older Adults

**DOI:** 10.1093/geroni/igab046.3498

**Published:** 2021-12-17

**Authors:** Juliana Souza-Talarico, Siomara Yamaguti, Adriana Dutra, Daniel Apolinario

**Affiliations:** 1 The University of Iowa, Iowa City, Iowa, United States; 2 Hospital do Coracao HCor, Hospital do Coracao (HCor), Sao Paulo, Brazil; 3 Hospital do Coracao HCor, Hospital do Coracao HCor, Sao Paulo, Brazil

## Abstract

Considering the limited evidence regarding the factors that contribute to long-term consequences after hospitalization of older people, we analysed the relationship between cognitive performance and hospital-associated complications (HAC). One thousand, three hundred Individuals aged 60 and older (mean age 82.3, 53.3% female), not assigned to palliative care and admitted in medical and surgical wards from a private hospital, were followed up from admission to 30 days after discharge. HAS was evaluated using a multicomponent measure that combines 12 hospital-associated complications (delirium, functional decline, falls, pressure injuries, bronchoaspiration, non-planned ICU transfer, physical restraints, hospital stay > 30 days, death, long-term care transfer, and readmission). Cognitive performance was assessed using the "10-point cognitive screener (10-CS)", which combines temporal orientation, category fluency, and word recall evaluation.

**Results:**

Overall, 464 (35.7%) participants had one or more HAC during their admission. Patients with HAC showed lower 10-CS scores than those with in HAC (p <0.001). Adjusting for sociodemographic data, medication, chronic diseases, delirium screening, functional performance, each 10-CS point decreased the HAC changes by 19.2% (odds ratio = 0.808; 95% CI = 0.660 – 0.990).

**Conclusion:**

These findings show that low cognitive performance was significantly associated with the risk of developing HAC during hospitalization and within 30 days after discharge. That evidence forms the critical foundation for the next steps towards validating the accuracy of these models in predicting vulnerability to HAC and developing screening tools to be used at the point of care.

